# Conservative care as a treatment option for patients aged 75 years and older with CKD stage V: a National survey in the Netherlands

**DOI:** 10.1007/s41999-018-0031-9

**Published:** 2018-02-12

**Authors:** Christopher Susanto, J. Kooman, A. M. Courtens, C. J. A. M. Konings

**Affiliations:** 1 Department of Internal Medicine, Section Nephrology, Elkerliek Hospital, Wesselmanlaan 25, 5707 Helmond, The Netherlands; 2 Department of Internal Medicine, Section Nephrology, Maastricht University Medical Center, Postbus 5800, 6202 AZ Maastricht, The Netherlands; 3 Expertise Center of Palliative Care, Maastricht University Medical Center, P. Debyelaan 25, 6229 HX Maastricht, The Netherlands; 4 Department of Internal Medicine, Section Nephrology, Catharina Hospital, Postbus 1350, 5602 ZA Eindhoven, The Netherlands

**Keywords:** Chronic kidney disease, Older patients, Renal replacement therapy, Conservative care

## Abstract

**Background and objectives:**

Conservative care for patients aged 75 years and older with CKD stage 5 as a treatment option besides dialysis was proposed officially in the Netherlands in October 2016. This national survey showed the current implementation of this option in Netherlands nephrology departments.

**Design, setting, participants and measurement:**

A web-based survey was sent to medical managers of 60 nephrology departments in the Netherlands in August 2016.

**Results:**

Twenty-one medical managers (35%) completed the survey. The term “conservative care” is frequently used and well known. The estimated number of patients in whom the decision for maximal conservative care was made in 2015 was 310 of 2249 patients with CKD stage 5 age 75 years and older (range 5–50 patients per department). 164 patients became symptomatic and received no dialysis. There is no official registration for this treatment option and patient category. The practice patterns vary widely. Only one of 21 respondents reported a conservative care outpatient clinic. Formal training or education regarding conservative care is not available in most of departments. 95% of respondents discussed this treatment option with their patients. General practitioners are always being informed about their patient’s decision. Their main role is providing or organizing palliative care support at the end of life and discussing advance care planning. Most respondents (86%) considered to include their patients in a prospective multicentre observational study, conservative care versus dialysis.

**Conclusions:**

Conservative care as a treatment option for patients with CKD stage 5 aged 75 years and older is well established. The practice patterns are varied in the Netherlands. Follow-up studies are needed to see whether the new multidisciplinary guideline facilitates harmonization of practice pattern. Funding is needed to optimize the implementation of conservative care.

## Introduction

The last decades have seen a marked growth in the rate of dialysis initiation in older patients with ESRD in all countries. The intensity of medical care for this particular group of patients is major. At the same time, poor survival and diminished quality of life for patients treated with dialysis, particularly older individuals, becomes an important health issue. Therefore, the decision to pursue dialysis or conservative therapy is more challenging than in other age groups [1–4].

Conservative care as an option of active treatment is increasingly being offered for the patients aged 75 years and older with CKD stage 5 in the USA [5], the UK [6], Canada [7], Australia [8], Europe [9] and Asia [10, 11].

The Renal Physicians Association (RPA) and the American Society of Nephrology (ASN) recommended to offer conservative or palliative care to all ESRD patients who suffer from burdens of their disease [5]. These include patients who have chosen not to start dialysis or withdraw from dialysis, but also patients who chose to undergo or remain on dialysis. This guideline recommends that a multi-professional team with expertise in renal conservative care should be involved in managing the physical, psychological, social and spiritual aspects of treatment for these patients, including end-of-life care.

In October 2016, the guideline about the choice of renal replacement therapy and conservative care was introduced in the Netherlands for professionals and patients [12]. At present, there are no data about the practice pattern of delivery of conservative care in the Netherlands.

In this paper, we present the result of a national survey of practice patterns and the implementation of conservative care as a treatment option for patients aged 75 years and older.

## Materials and methods

We conducted a web-based survey of conservative care as a treatment option for patients aged 75 years and older with CKD stage 5.

The survey was emailed to 60 medical managers of nephrology departments in the Netherlands. The invitation was sent to them in August 2016. There was no input from commerce or industry and no incentive was offered to respondents. 8 weeks after survey distribution, we sent one additional reminder to nonrespondants. All responses were kept confidential. The survey was approved by Medical Ethical Review Committee of Elkerliek Hospital.

The content of the survey was based on an earlier national survey in the UK which was published in 2014 [13]. The first part of the survey assessed how the nephrology department is organized with regard to patients with CKD in general. The second part concerned the availability of an alternative to dialysis, the development and implementation of conservative care. The third part consisted out of three components: (a) how is conservative care discussed with the patients, (b) the co-operation with general practitioners regarding with this care and (c) the evaluation of conservative care in the nephrology department. The last part assessed the development and presence of end of life care (Advanced Care Planning: ACP). Some of the components of the survey were adjusted for the situation in the Netherlands. Data were analyzed using descriptive statistics and were summarized as mean or percentages.

## Results

Thirty-one medical managers (51%) responded to the survey, only twenty-one completed the survey. Ten incomplete responses are not included in the analysis. Two (9.5%) respondents represented nephrology departments of academic hospitals and one respondent (4.8%) represented a private dialysis centre. 85.7% of responses came from medical managers of general hospitals.

This study reported there were 2249 patients with CKD stage 5 aged 75 years and older in 2015 (range 20–400 patients in participating centres). There were approximately 310 patients on conservative care (range 5–50 patients per department). 164 of 310 patients became symptomatic (range 2–40 patients in participating centres) and did not receive dialysis.

Each respondent indicated that these numbers were estimates since there was no official registration system.

### Organization of nephrology department for CKD patients in general

The median of full time equivalent (FTE) nephrologists working at the Nephrology department was 4.6 (range 1.8–10 FTE). In all Nephrology departments, a multidisciplinary team is available to manage patients approaching renal replacement therapy. This multidisciplinary consultation takes place once a week in most nephrology departments (86%). The team consists of dialysis nurse, nephrologist, (nephrologist trainees), dietician and social worker. Palliative care consultants were never involved.

95% of the respondents mentioned the presence of a pre-dialysis clinic in their department. 50% of respondents did not mention a collective pre-dialysis clinic. The main reason for refraining from a collective pre-dialysis clinic is to keep long-term continuity of care by the same physicians. A pre-dialysis nurse has an important role to give information about renal replacement therapy. Pre-dialysis education is mostly delivered via general DVD education and written material to take home. There is flexibility to allow extra education time for those who need it.

Ten nephrology departments organized a course called “living with CKD”, thrice a year. Nine nephrology departments organized a pre-dialysis education day, during which all topics of CKD and treatments are presented including the option of conservative care.

### Availability of an alternative to dialysis, the development and implementation of conservative care

All respondents reported experience with patients who decided not to do dialysis even if they were symptomatic. This group of patients is followed in pre-dialysis clinics. Only a small percentage of patients are referred back to general practitioners.

The term of conservative care is being used when referring to the care of patients with CKD stage 5 where a decision is made not to do dialysis.

52% of the respondents indicated that there is no written guideline for how to manage patients on conservative care.

76% of the respondents (16 nephrology departments) do not have staffs who specialize in conservative care. Only five nephrology departments (24%) do have a single person (nephrologist) who is primarily responsible for conservative care. Formal training or education regarding conservative care is not available in most departments. 47% of respondents reported that they are still waiting for the guideline and they are not convinced about the necessity of specific education or training. Other reasons were as follows: lack of time (40%), do not need formal training as conservative care is an ingrained culture in the department (20%), lack of funding (13%) and nephrologist’s lack of interest in the training (13%).

**Fig. 1:**
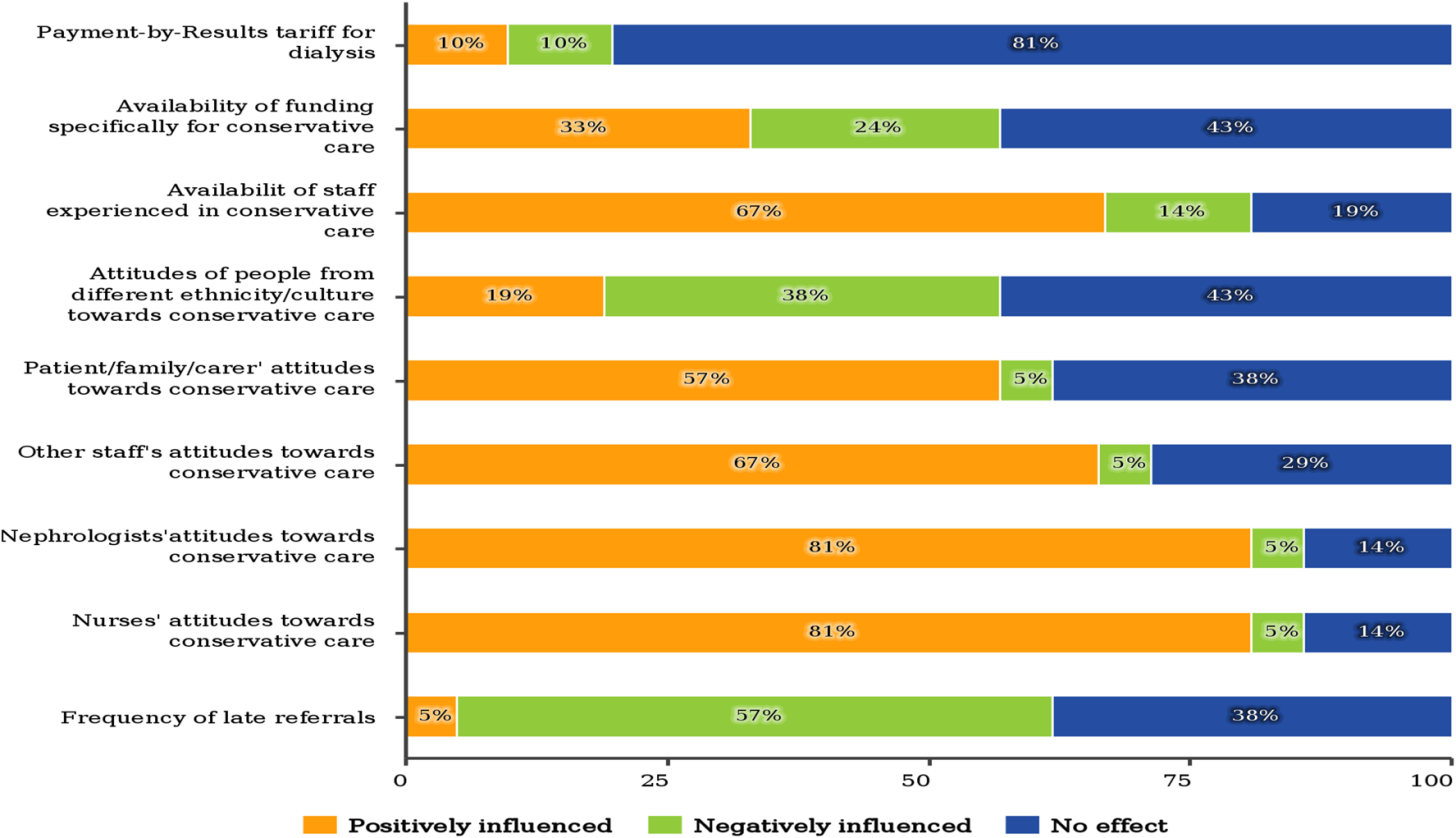
The factors influence the development of the conservative care programme

The attitude of nephrologist, nurse and other unit staff toward conservative care influence positively the development of the conservative care program. Negative influences for the development of this program are obviously late referrals, lack of time and limited availability of staff for setting up this program, while payment for dialysis has only a small effect (Fig. 1).

**Fig. 2:**
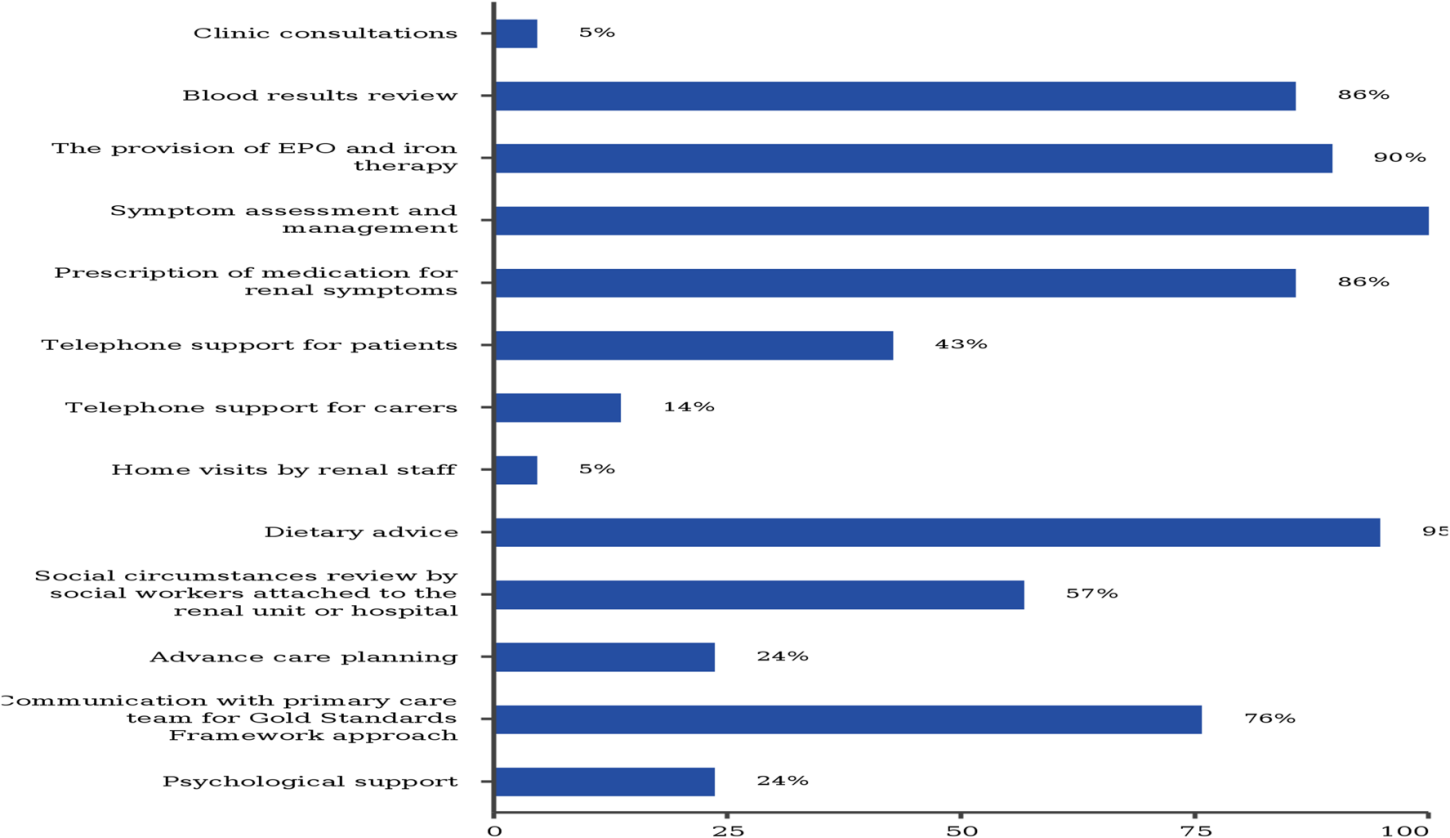
The key components of conservative care provided to patients in nephrology department

Furthermore, there is no clinic available exclusively for patients with CKD stage 5 on conservative care. Only one nephrology department runs a conservative care clinic once per month in their nephrology outpatient clinic. Symptomatic patients are seen monthly, while asymptomatic patients are usually seen once in 3 months. The key components of conservative care provided to patients are mostly symptom assessment and management (100%), social circumstances review by social workers attached to the department (57%) and small percentages of psychosocial support for patients and caregivers, including advanced care planning (Fig. 2).

There is no additional funding dedicated to providing and developing conservative care.

### Discussing conservative care with patients

**Fig. 3:**
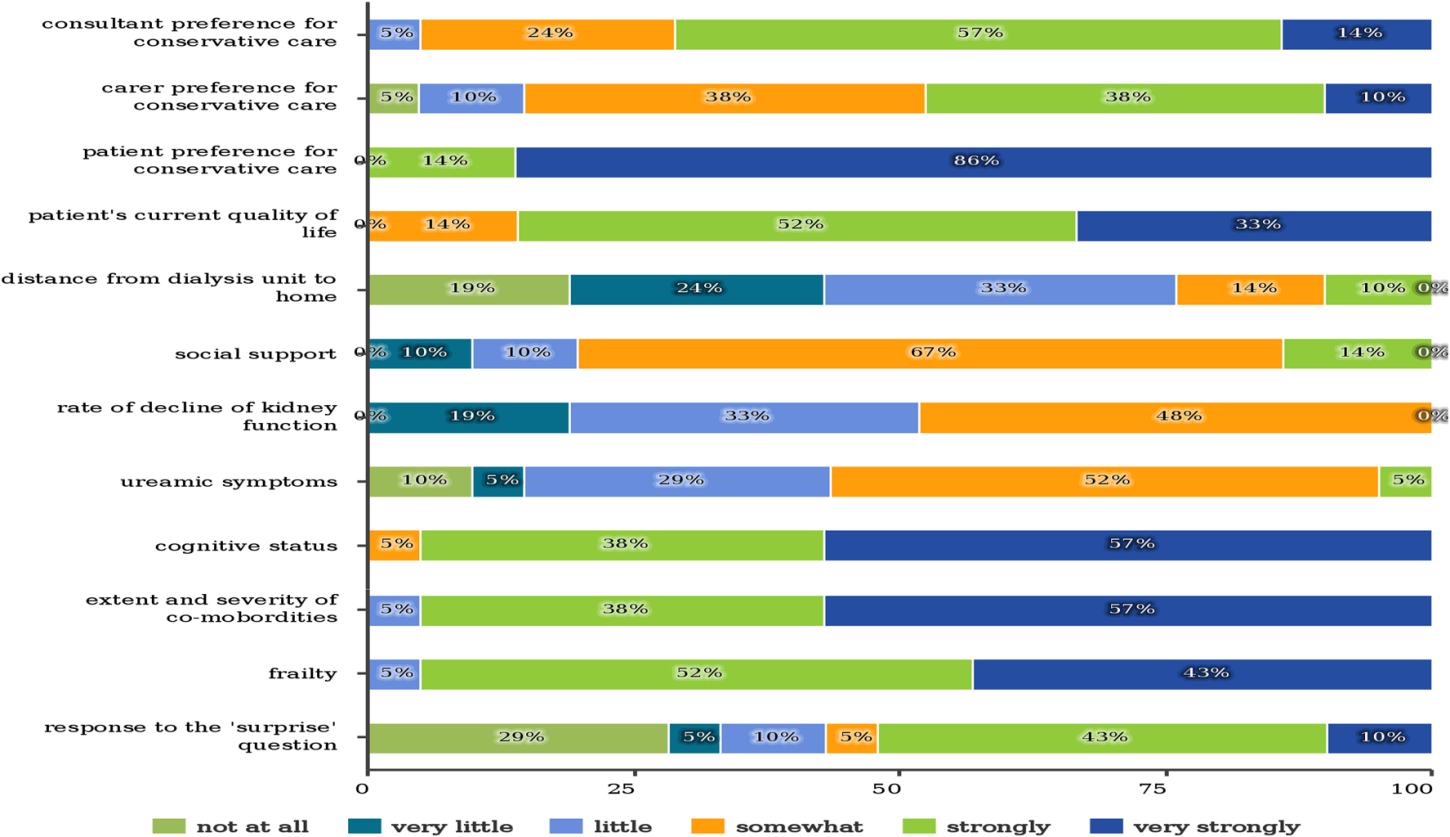
Factors are likely to influence nephrologist when contemplating the suitability of conservative care for a patient

The option of conservative care is mostly discussed with all patients with CKD stage 5 aged 75 years and older (95%). The following factors strongly affect staff when contemplating the suitability of conservative care for a patient: patient preference, cognitive status, extent and severity of comorbidities, frailty and patient’s current quality of life (Fig. 3).

Most of the time the discussion took place when the patients were referred to a pre-dialysis clinic (48%). Only 24% of the respondents use practical tools when they discuss the option of conservative care with patient. All of them use booklets/handouts from the Dutch Kidney Foundation (Nierstichting). Family and caregivers were involved in this process. They are encouraged to attend the dialysis clinic with the patient, are also invited to the patient education day, and are involved in home visits. Most important, they are involved when the patient is revisited regarding a conservative care decision. Review of the decision takes place at the request of patient or caregivers. In certain circumstances and due to some reasons, the decision not to go on dialysis will be made by the nephrologist in charge with input from other consultants (100%). All of the information is recorded only in the medical file (100%). 67% of the respondents reported that patients who decide not to have dialysis changed their decision. This happened because of the patient’s wishes such as a wish to attend a special event (wedding, birth, graduation, etc.), their severe symptoms which cannot be controlled with conservative treatment and fear of death.

### Co-operation with general practitioners

The general practitioner (GP) is always being informed about the patient’s decision not to have dialysis (100%). The nephrologist gives written and verbal advice regarding the treatment of CKD stage 5 patients receiving conservative care.

Further collaboration between nephrologist and GP has been established by “patient preference”. Some patients preferred primarily to be under the care of a nephrologist with little GP involvement and some preferred to receive care from their GP with nephrologist’s collaboration.

The main role of GP in the management of CKD stage 5 patients receiving conservative care is to provide and/or to organize palliative care support at the end of life and to discuss advance care planning with patients.

### The evaluation and future development of conservative care

Only one respondent (5%) reported that the quality of conservative care is regularly evaluated. This respondent noted information such as hospitalization, patient and caregiver’s experience with conservative care and evaluation with GP.

**Fig. 4:**
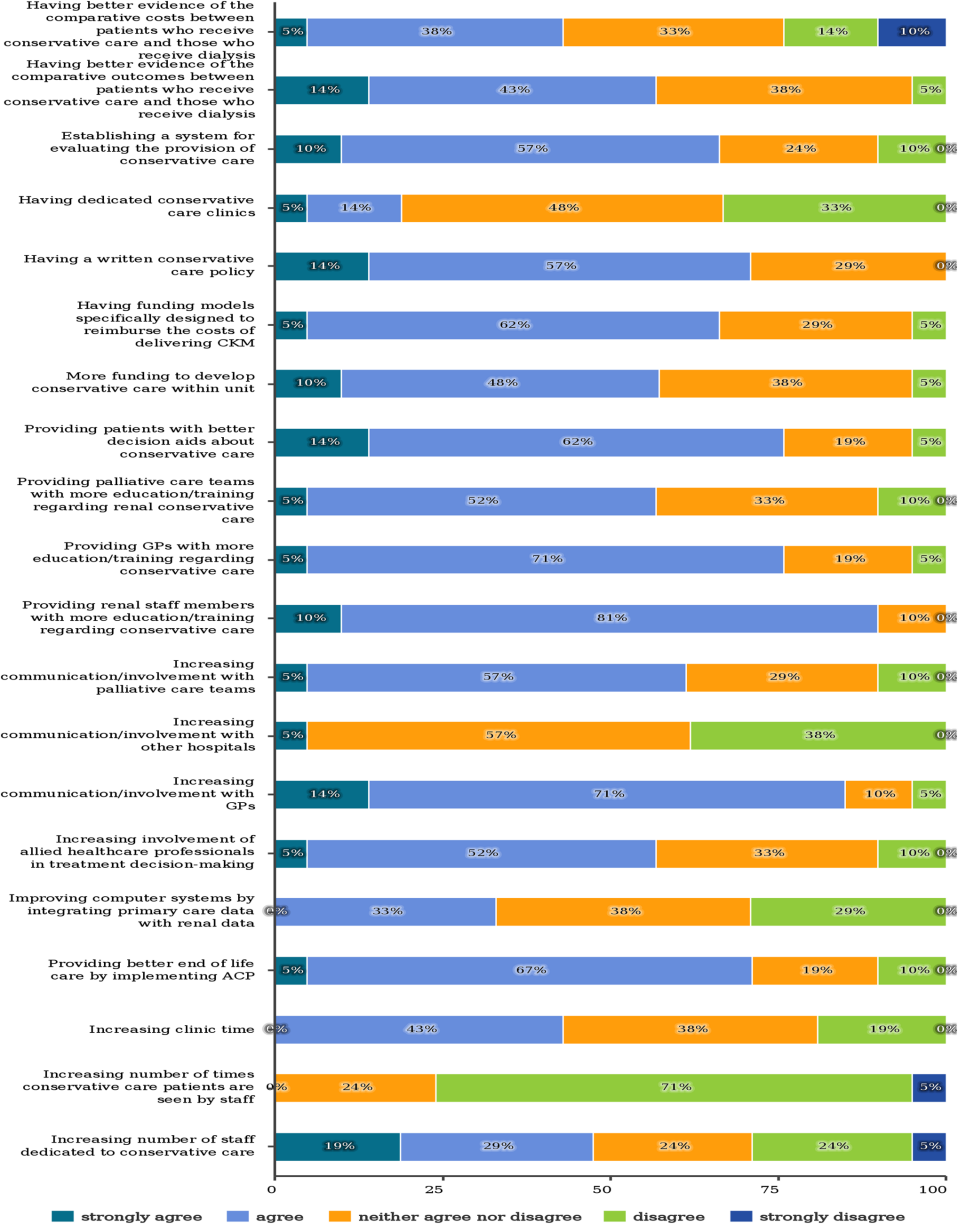
Factors could help improve the provision of conservative care in nephrology department

The factors reported to be most important to improve the development of conservative care were providing renal staff members and general practitioners with more education and training, increasing communication and involvement with general practitioners, providing patients with better decision aids and providing better end of life care by implementing ACP (Fig. 4).

Only 57% of the respondents agreed that having better evidence of the comparative outcomes between conservative care and dialysis would help improve the development of conservative care.

18 respondents (86%) would consider entering a 75 year and older with CKD stage 5 into a prospective observational study of conservative care versus dialysis. Seven of the 18 respondents reported their willingness to participate in such a trial.

### The development and presence of end of life care (ACP)

57% of respondents reported that they do not have a written guideline for end of life care. 14% of respondents use a local guideline and 29% of respondents claimed that their local guideline was in preparation. Only 10% of respondents identified conservative care patients approaching end of life through use of a local register, the rest reported it in the patient’s medical file. The following factors strongly influenced a decision to add a patient to the end of life register: frequent hospitalization, symptoms, quality of life, frailty, comorbidities and GFR level.

ACP in end of life care is used by 5 of 21 respondents (24%). Renal nurse, nephrologist and social worker are usually involved. General practitioners were not involved initially in this process. In these departments (24%), the staff received special training for palliative or end of life care for CKD patients. When patients who receive conservative care, approach end of life, appeal is made to specialist palliative care services in hospitals (62%), primary care team (57%) and specialist palliative care from local hospice (33%).

Most of these services are being given at home (84%), within hospital as in patients and at hospice where patients are admitted at end of life (68%).

## Discussion

Conservative care as a treatment option for older patients with CKD stage 5, most of whom have multiple comorbidities, has been proposed and discussed in different countries [1, 3, 4, 6–8, 10, 14]. Still, there is no uniformity about the implementation of this option in daily practice.

Recently, in October 2016, a formal guideline about the choices of renal replacement therapy (RRT) and conservative care was introduced in the Netherlands. An important recommendation of this guideline is that the age of the patient should not be used as a primary criterion for making a proper choice about RRT or conservative care, but that also comorbidities, performance status and quality of life should be taken into account in the decision-making process.

To our knowledge, this is the first national survey which explores and describes the current scale and pattern of implementation of conservative care for older patients who choose not to have dialysis.

In the Netherlands, multidisciplinary pre-dialysis care is well established. Treatment options are discussed with the patient and relatives or caregivers.

All respondents start to provide information about renal replacement therapy for patients when they are referred to the pre-dialysis clinic (e-GFR were ≤ 20 ml/min per 1.73 m^2^). All of them use handouts from the Dutch Kidney Foundation (Nierstichting). Family and caregivers are involved in the process. Most of respondents mentioned conservative care as a treatment option when they discussed renal replacement therapy with patients aged 75 years and older. Few discussed this option when patient’s e-GFR is lower than 15 ml/min per 1.73 m^2^. In Australia and in UK, patients received information about treatment options when their e-GFR was < 15 ml/min per 1.73 m^2^ and was about 20 ml/min per 1.73 m^2^, respectively [13, 15]. This survey showed still there is no uniformly accepted period to discuss the option of treatment with patient. This is because of uncertainty and unpredictability of the illness trajectory. Many patients with CKD have a nonlinear GFR trajectory or a prolonged period of no progression [16].

This survey emphasized that a careful assessment of disease progression from each individual patient is necessary to decide when to discuss conservative care as a treatment option.

Conservative care as a treatment option is well accepted in the Netherland’s nephrology department but the implementation of this option is quite diverse. For examples, in the Netherlands, there is no formal clinic dedicated to conservative care. Most of the departments provide their conservative care services in the pre-dialysis clinic. However, most departments do not have staffs who have specialized in conservative care. This finding is in stark contrast to the high percentage (50%) found in the UK [13].

Furthermore, there is no formal training or education available regarding conservative care. Nephrologist’s lack of time and interest seem to be one of the reasons for not having staffs who have specialized in conservative care. While the lack of funding has not been named as an important reason, however, over 50% of the respondents reported more funding could help positively the development of the conservative care program. In our survey, the majority of respondents believe that a positive attitude of nephrologist, nurse and other unit staff toward conservative care will stimulate the development of a conservative care program. Therefore, we believe that the KDIGO’s proposal (Kidney Disease Improving Global Outcomes) about a detailed and specific definition for kidney conservative care, which encapsulates the full range of management and interventions involved, should be promoted [17].

The national data (Renine) showed there were 16,277 patients in 2015 whom received renal replacement therapy of which 2983 patients were 75 years and older. 592 patients received kidney transplantation and 2391 patients received dialysis. These data were captured prospectively, contrary to the numbers of patients receiving conservative care. These numbers could be small in comparison with the number of patients aged 75 years and older receiving renal replacement therapy. Neither our survey nor the previous survey in the UK showed the exact numbers of patients choosing conservative care as a treatment option. Meanwhile, this group of patients appears to have grown exponentially the last decade. Formal registration for conservative care is necessary for further studies to improve better services and care for this group of patients.

In the Netherlands, patients with CKD stage 5 have a special tariff code. This tariff code will change when the patient receives renal replacement therapy. But the tariff code will not change when conservative care as a treatment is chosen. The lack of a tariff for conservative care is considered as one of the most important obstacles to developing a specific conservative care program. Considering that conservative care could last up to a few years, it involves more than just symptoms management but also guidance for end of life and advanced care planning (ACP).

All respondents informed the general practitioner about the patient’s decision. Further collaboration between nephrologist and general practitioner has been established by patient preference. Most respondents agree that nephrologist and general practitioner should share the care and work closely together to give an optimal guidance for patients in this stage of their life and stressed the need for a conservative care team consisting of nephrologist, renal nurse, social worker, general practitioner and or specialist palliative care.

This team has a central coordinating role in the end-of-life phase and need renal specific training such as the training in advance communication skills tailored to advanced kidney disease [13].

Our survey has received only a moderate rate of response. Some data are estimates and so should be regarded with caution, such as the number of patients aged 75 years and older and also the number of patients who chose conservative care as a treatment option.

In summary, this survey showed the following situations: First, conservative care is emerging as a treatment option for older patients with CKD stage 5 in the Netherlands. There is no disagreement about terminology. But a clear definition of conservative care is necessary. Second, the content and prescription of treatment and the method of guidance varies between clinics. Third, there is a large unmet need for identification, registration and provision of funding to facilitate the development of a conservative care program.

Training and education about this specific care are needed for the nephrology team to improve the quality of care, specifically how to discuss and address conservative, palliative and end of life care. Furthermore, a good collaboration with the general practitioner and or palliative care specialist should be stimulated.

Follow-up studies are needed to see whether the new multidisciplinary guideline facilitates harmonization of practice pattern.
